# Effect of Photobiomodulation on Crestal Bone Density Around Dental Implants: A Case Report

**DOI:** 10.7759/cureus.63546

**Published:** 2024-06-30

**Authors:** Ashika Singhania, Anjali Bhoyar, Surekha A Dubey, Tanvi Jaiswal

**Affiliations:** 1 Prosthodontics, Sharad Pawar Dental College and Hospital, Datta Meghe Institute of Higher Education and Research, Wardha, IND

**Keywords:** dental implants, bone density, compromised bone, implant stability, low level laser therapy, photobiomodulation

## Abstract

Dental implants are becoming a necessary component of the dental profession. The first bone resorption at the implant surface has an impact on implant success. Bone alterations surrounding the implant are a significant factor in determining the implant's effectiveness. Reducing the loss of peri-implant crestal bone has been a constant goal. In dental implantology, several procedures are carried out to improve implant stability and the healing of the bone. The ability of photobiomodulation techniques or low-level laser therapy (LLLT) to speed up osseointegration by inducing cellular metabolism and stimulating tissue healing has made them popular. This case study details the implant loading in a patient treated with photobiomodulation to achieve implant stability and who has compromised bone type or D4.

## Introduction

Patients with dentition anomalies are drawn to dental implants because of their exceptional looks, high success rates, and functional features. However, because of a deficiency in osseointegration, many patients continue to face the risk of implant failure. Osseointegration can be influenced by several things. The process of osseointegration is aided by several physical, chemical, and biological elements, one of which is "photobiomodulation therapy (PBMT)," also known as low-intensity laser therapy [1]. For over three decades, low-level laser treatment, or LLLT, has been used in the medical industry with no documented side effects. It is defined as red beam or near-infrared laser therapies with low energy density and output power that do not raise normal body or tissue temperature at wavelengths between 500 and 1,200 nm. Consequently, it has non-thermal and biostimulative properties [2]. Through the induction of cytokines and growth factors that drive fibroblast migration and proliferation, it accelerates angiogenesis, improves mesenchymal cell differentiation into osteoblasts, and increases the osteoblasts' adhesion to the surface of titanium implants, all of which contribute to the process of bone regeneration. Additionally, studies have shown that LLLT at the molecular level increases the creation of bone nodules while improving the mechanical strength of the interface between the implant and the bone [3]. Implant sites frequently lack the necessary ideal conditions for either sufficient or high-quality bone. According to preliminary procedures, three to four months are needed for healing after implant placement. This process takes longer and may take up to six months since the maxilla and posterior mandible have bone which is cancellous in nature. It is consequently necessary to develop methods to enhance the amount and density of bone and encourage bone regrowth surrounding implants. The LLLT treatment is used to increase the main stability of dental implants in low-density bone, which speeds up the healing process [4]. This case report describes implant loading in a patient having D4 or compromised bone type treated using photobiomodulation to achieve implant stability and prosthetic rehabilitation.

## Case presentation

Patient information

A 45-year-old male patient presented to the Department of Prosthodontics and Crown & Bridge with a complaint of missing teeth in the lower left back region of the jaw for the past two years, wanting a fixed replacement. The patient had a medical history of diabetes and was under medication currently.

Clinical findings

Oral examination revealed missing teeth #35 and #36. The edentulous ridge in relation to #35 and #36 appeared resorbed.

Investigations

**Figure 1:**
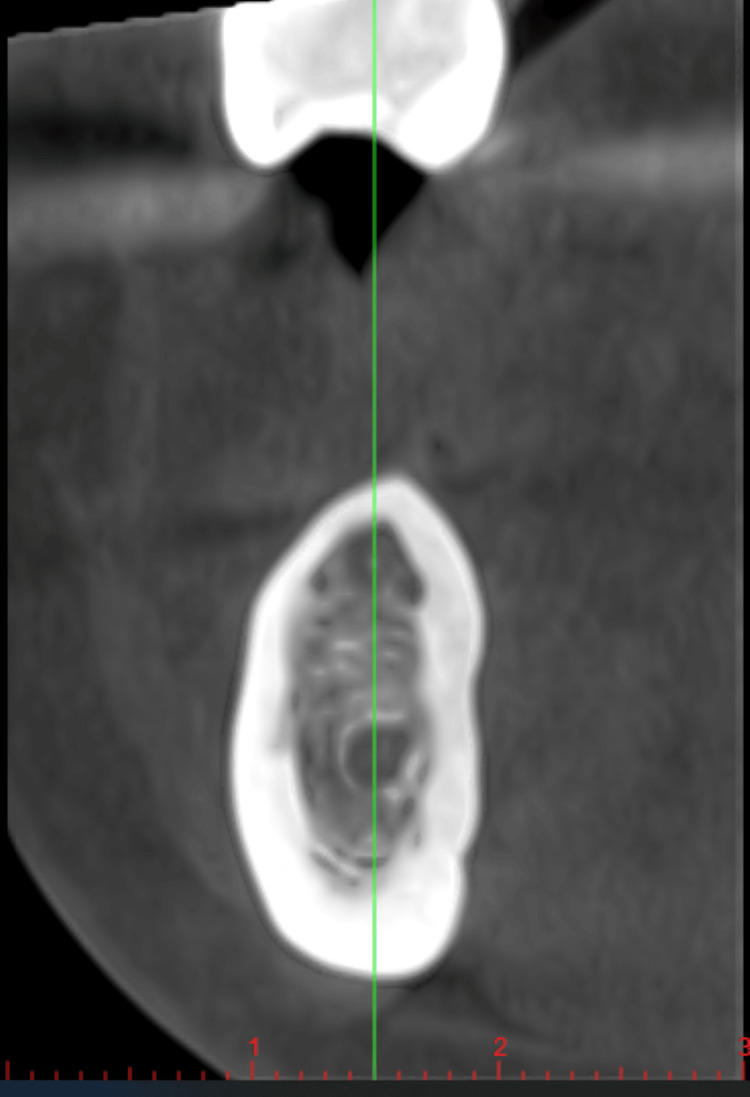
Cone beam computed tomography of edentulous area

Cone beam computed tomography (CBCT) was taken for the patient, and the bone density measured was 320 HU, which categorizes the bone into D4 bone type or compromised bone (Figure 1).

Treatment plan

Diagnostic impressions were made using irreversible hydrocolloid impression material. Various treatment options were discussed with the patient including rehabilitation using a tooth-supported fixed dental prosthesis, conventional implants with bone augmentation, and conventional implants with photobiomodulation therapy. Upon obtaining the patient's consent, it was decided to proceed with a conventional implant with photobiomodulation therapy.

The mandibular posterior edentulous site was implanted using a standard surgical technique. Asepsis was strictly maintained during the procedure. Local anesthesia was administered using a 2% lignocaine hydrochloride solution containing adrenaline at a ratio of 1:100,000. Following a mid-crestal incision, a full-thickness mucoperiosteal flap was lifted, and osteotomy was performed with abundant normal saline irrigation. A direction indicator was utilized to ensure that the fixture was oriented correctly before drilling the insertion location. Next, an Osstem implant fixture measuring 3.8 mm in diameter and 11 mm in length was inserted, achieving good primary stability (20 Ncm torque).

**Figure 2:**
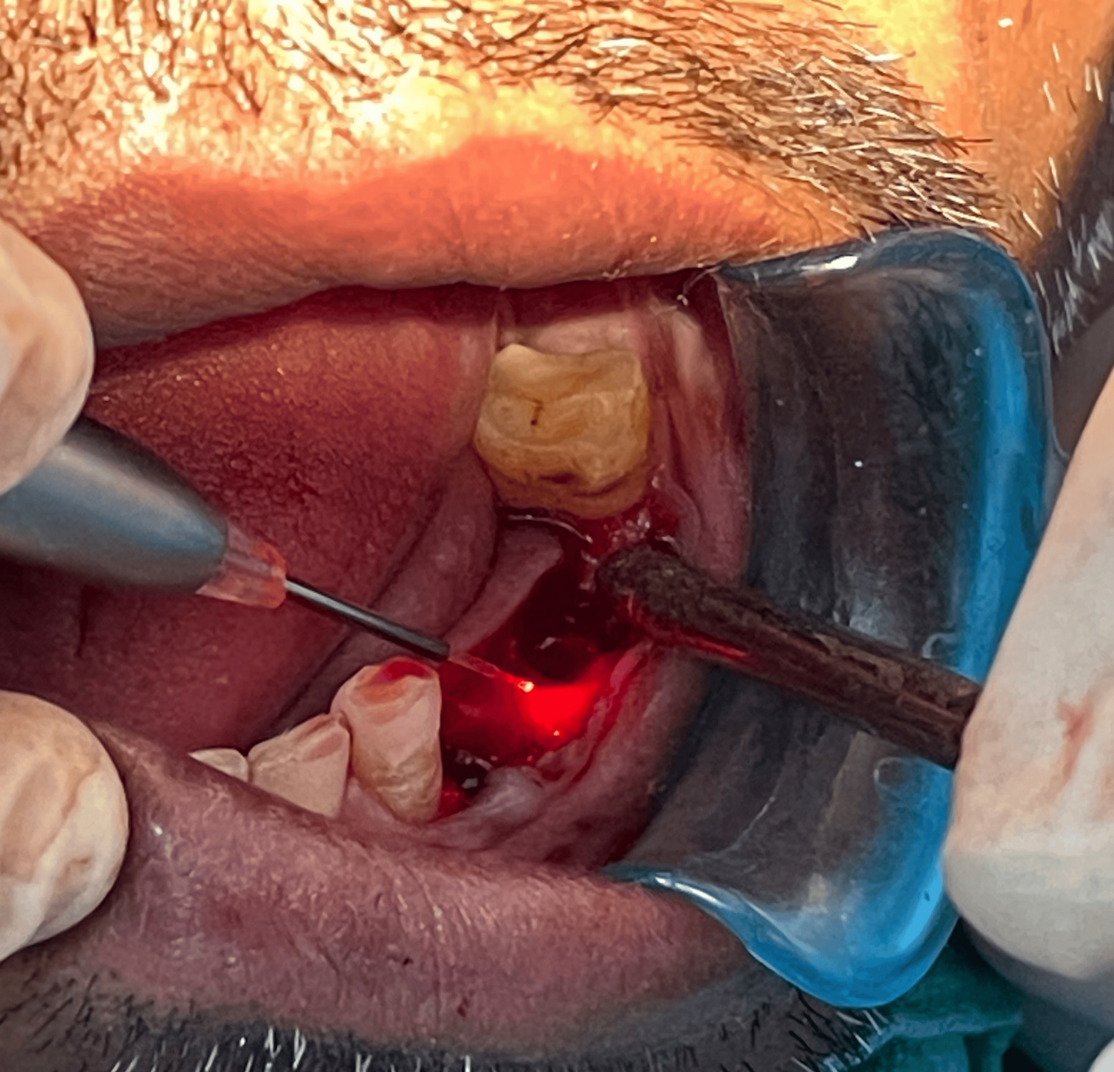
Buccal and palatal sides subjected to photobiomodulation

**Figure 3:**
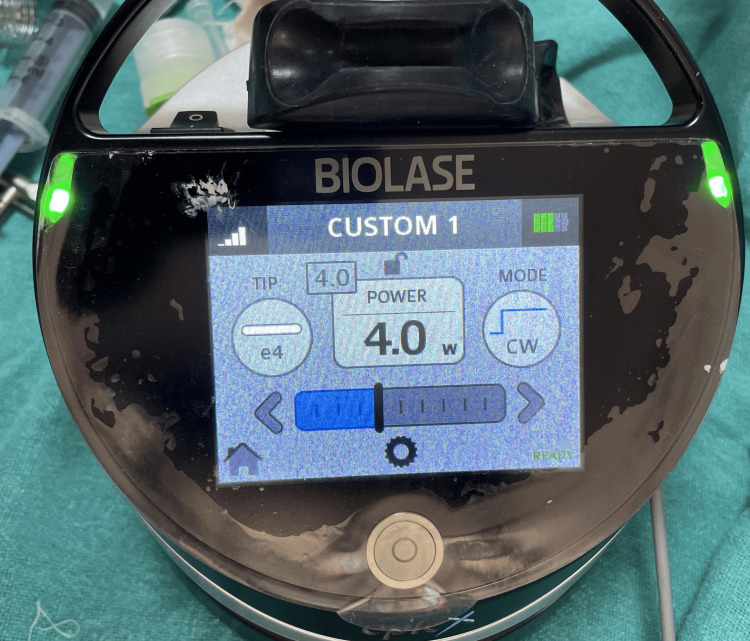
Biolase laser specifications

Photobiomodulation therapy was administered to the patient before suturing. A clinician in a separate room used the diode laser (epic10, BIOLASE, Inc., Irvine, CA) to emit radiation. Both the patient and the clinician wore protective eyewear as part of the safety measures. The laser's specifications were a diode laser operating in continuous wave mode at a wavelength of 940 nm, an output power of 100 mW, a spot area of 0.2826 cm^2^, and an average power density (irradiance) of 354.6 mW/cm^2^. For 40 seconds, tissue probes in contact with the mucosa on the buccal and palatal sides were subjected to laser radiation (corresponding to 14.18 J/cm^2^ energy density) on each side (Figure 2). The energy used in each session was 4 J on the buccal and palatal implant sides, for a total of 8 J (Figure 3).

**Figure 4:**
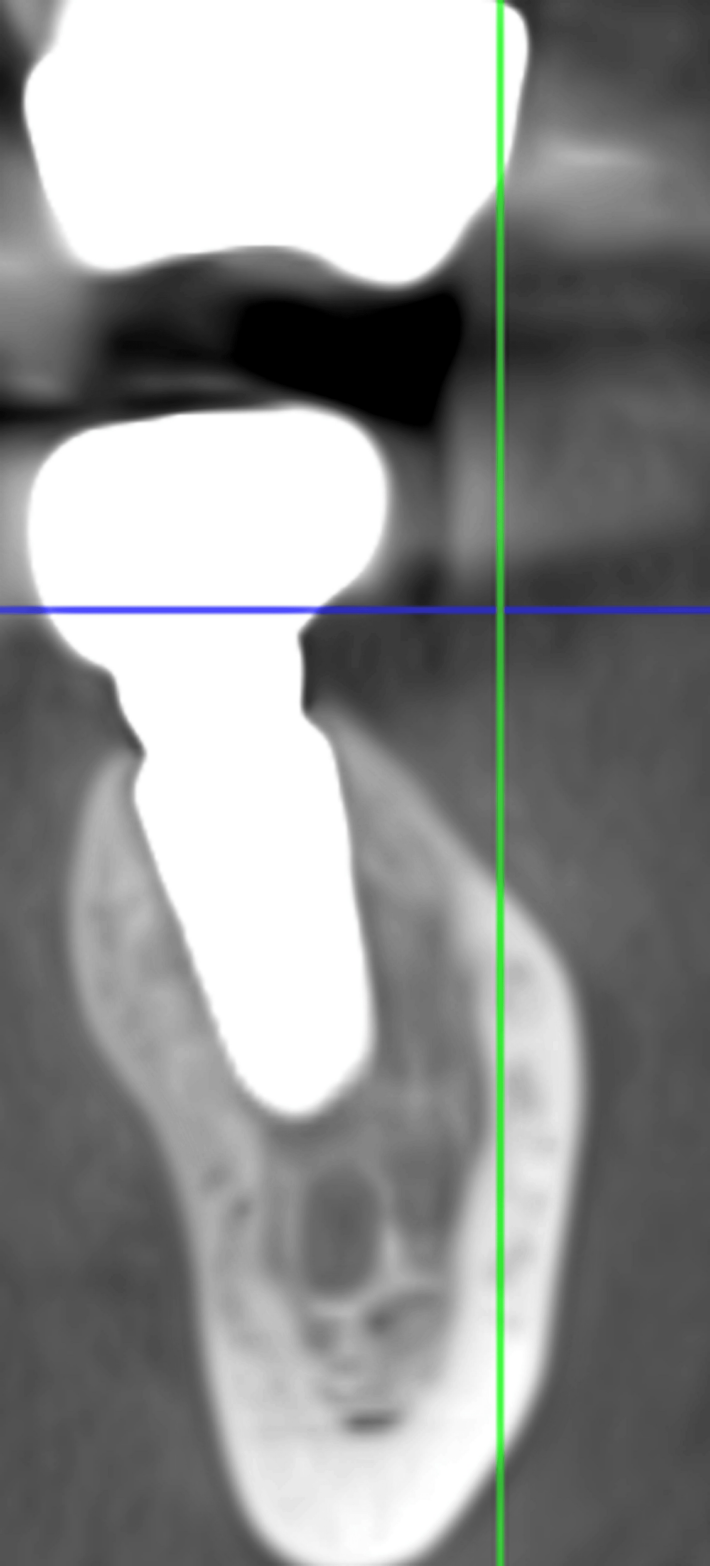
Cone beam computed tomography (CBCT) at three months

After the procedure, there were follow-up laser treatments at 2, 4, 6, 8, 10, and 12 days. The total dose was 56 J (the sum of the energy from all seven sessions). Following surgery, each patient was provided 625 mg of amoxicillin and clavulanic acid orally every eight hours for seven days, beginning one hour before the procedure, 50 mg of diclofenac sodium orally every eight hours for three days, and three times a day for seven days of mouthwash containing 0.12% chlorhexidine. The patient was recalled after three months, and a repeat CBCT was taken. The CBCT analysis in the region of 35 and 36 was 800 HU, which showed an improvement in implant stability and quality of bone (Figure 4).

**Figure 5:**
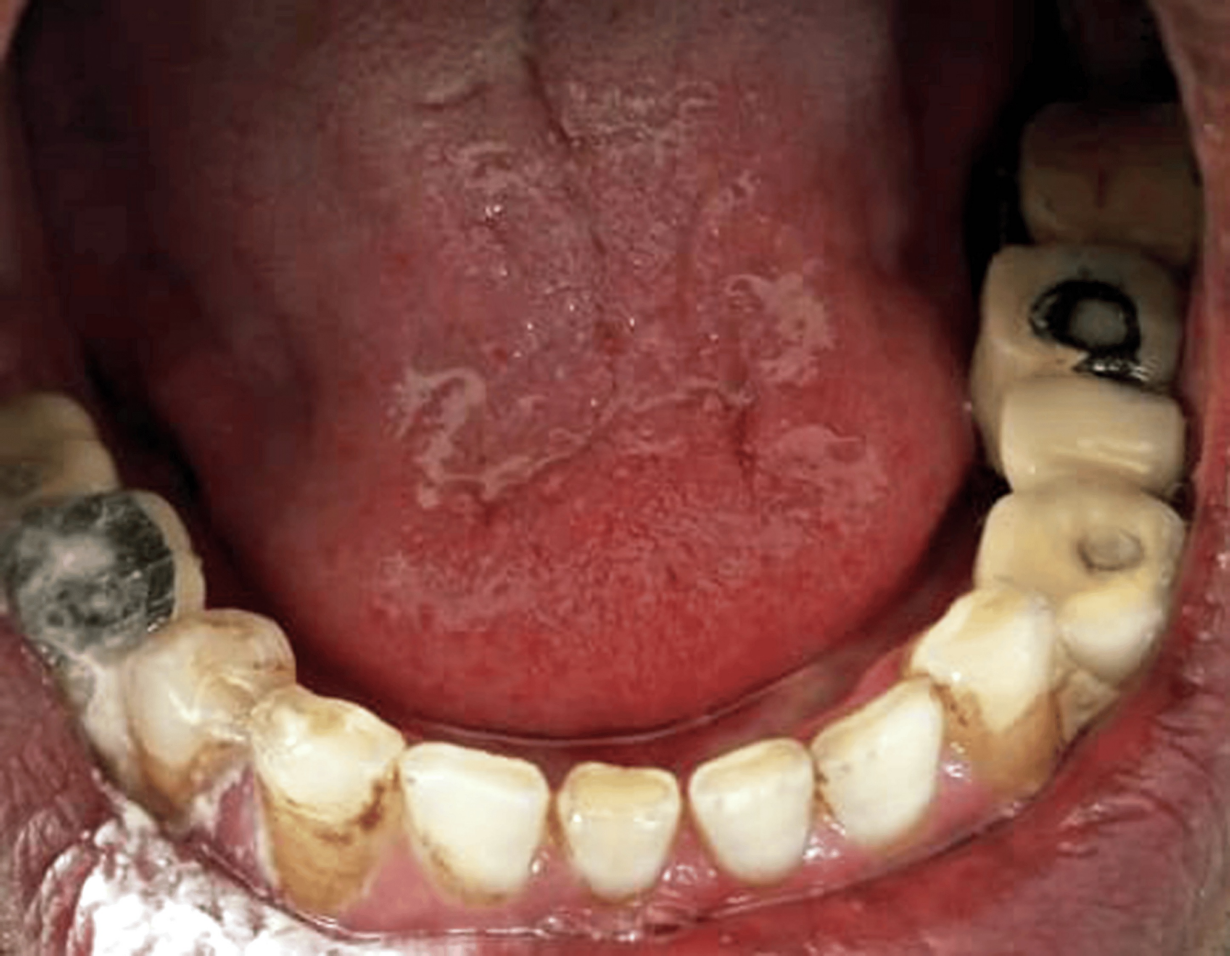
Postoperative image

The prosthetic phase was planned, and metal-ceramic crowns were fabricated for the patient after the second stage of surgery (Figure 5).

Follow-up and outcome

Follow-up was done after three months, and the patient was evaluated for the prosthesis, which was found to be satisfactory.

## Discussion

In the present case, photobiomodulation therapy was given to a patient with compromised bone as it has proven effects. Light-emitting diodes or lasers are applied to particular tissues during LLLT; it has been suggested that this kind of light radiation can change biological processes. Research indicates that the LLLT may have a physiological impact on osteoblasts by increasing mitochondrial activity, elevating adenosine triphosphate synthesis, and stimulating the production of reactive oxygen species. Increased osteoblast proliferation and differentiation may result from these biological reactions by activating diverse signaling pathways, for instance, those involving transcription factors and growth factors [5].

A study by Matys et al. suggests that the best way to achieve the best biological reaction is to use fluence between 1 and 10 J/cm^2^. In the current instance, the secondary implant stability might be increased with a dose per point of 4 J (8 J/cm^2^). Using CBCT, this case study also assessed how the LLLT affected bone density after the placement of dental implants. The enhanced Hounsfield units seen three months later demonstrate how effective LLLT using a 940 nm diode laser is at boosting bone density and secondary implant stability [6].

Cell components are less susceptible to LLLT because they are more prevalent in the initial phases of bone regeneration. Osteoblasts multiply during the primary cell-rich phase, and lengthier laser therapy cycles can effectively promote cell proliferation during this phase. Greater bone matrix deposition, calcification, and bone maturation are caused by a higher cell count. As a result, in our investigation for the first 14 days, every other day was the schedule for administering laser radiation, which seemed adequate in terms of both duration and interval [4].

The literature reports a wide range of low-level laser wavelengths, from 600 to 1100 nm. While photons in the near-infrared range (780-1100 nm) of the electromagnetic spectrum penetrate to a depth of 8-10 mm, those in the red area (600-700 nm) are only superficially absorbed. As tissue chromophores absorb and scatter light at near-infrared wavelengths less frequently, osteoblasts are better able to absorb laser energy. The Biolase laser, which has a wavelength of 940 nm and falls in the near-infrared region, is chosen for this case report [3].

The application of LLLT in dentistry has been extensively researched in recent decades. Studies in oral implantology have evaluated the application of LLLT to enhance primary stability in the initial phases of osseointegration. Applying photobiomodulation therapy to enhance implant stability can prove beneficial for the patients. The literature on this topic has numerous changes in laser settings and dosage. To find out how effective PBMT is, studies standardized on laser parameters should be conducted [7].

This case was followed up for six months, and the patient exhibited no bone loss and an excellent prognosis. This shows that with an accurate diagnosis, treatment planning, and proper knowledge of the available techniques, patients with compromised bone can also achieve improved results.

## Conclusions

By improving crestal bone levels and influencing tissue repair, the osseointegration of the implant into the bone may be accelerated by photobiomodulation with LLLT. Practitioners can improve implant success rates, patient outcomes, and satisfaction particularly in patients with impaired bone by optimizing photobiomodulation procedures and being aware of the possible advantages and limitations of this new therapy.
